# Melatonin Mediated Regulation of Drought Stress: Physiological and Molecular Aspects

**DOI:** 10.3390/plants8070190

**Published:** 2019-06-26

**Authors:** Anket Sharma, Bingsong Zheng

**Affiliations:** State Key Laboratory of Subtropical Silviculture, Zhejiang A&F University, Hangzhou 311300, China

**Keywords:** abiotic stress, plant stress physiology, oxidative stress, water deficit conditions, water stress

## Abstract

Drought stress adversely effects physiological and biochemical processes of plants, leading to a reduction in plant productivity. Plants try to protect themselves via activation of their internal defense system, but severe drought causes dysfunction of this defense system. The imbalance between generation and scavenging of reactive oxygen species (ROS) leads to oxidative stress. Melatonin, a multifunctional molecule, has the potential to protect plants from the adverse effects of drought stress by enhancing the ROS scavenging efficiency. It helps in protection of photosynthetic apparatus and reduction of drought induced oxidative stress. Melatonin regulates plant processes at a molecular level, which results in providing better resistance against drought stress. In this review, the authors have discussed various physiological and molecular aspects regulated by melatonin in plants under drought conditions, along with their underlying mechanisms.

## 1. Introduction

In the present era, water scarcity is one of the main environmental challenges for plants, which has negative impacts on their growth and development [1,2]. The reduction of water availability to plants causes physiological imbalances which ultimately reduces plant productivity [3]. The impact of drought stress on the physiological responses of plants is dependent upon the level of drought, its exposure time and the growth stage of plants [4]. Drought stress induces phytotoxicity by enhancing accumulation of reactive oxygen species (ROS) in the plant cells, which is mainly due to the imbalance between ROS generation and their scavenging [1,5]. Increased concentrations of ROS during drought conditions negatively affect the photosynthetic reactions by disrupting the photosynthetic apparatus, including reaction centers and chloroplast structures [6,7,8,9]. Furthermore, enhanced ROS accumulation favors the degradation of chlorophyll molecules and finally declines the photosynthetic performance of plants under water deficit conditions [10,11]. 

As plants are exposed to various biotic and abiotic factors, they possess an inbuilt system, known as antioxidant system, to regulate the biological processes under adverse environmental conditions. This antioxidant system is comprised of enzymatic and non-enzymatic antioxidants, which work in a systematic manner to control the levels of ROS in plant cells [12]. However, under severe stress conditions, such as high drought levels, this antioxidant system is disrupted, leading to an imbalance in redox homeostasis in plant cells [13,14,15]. 

Plant growth regulators are multifunctional molecules which are well known for their physiological functions in plants [16,17,18,19]. These molecules also play an important role in providing resistance to plants growing under abiotic stresses, such as heavy metals, temperature, pesticides and drought [20,21,22,23,24,25,26]. Melatonin is a growth regulator which also confers stress tolerance to plants growing under adverse conditions such as drought [6,27,28]. Melatonin regulates the biology of plants by modulating various physiological, biochemical and molecular processes and ultimately enhances resistance in plants to withstand drought conditions [29,30]. The regulation of photosynthetic machinery and the anti-oxidative defense system are the main physiological processes controlled by melatonin under water deficit conditions [31,32]. In the recent past, considerable research has been undertaken to explore the effects of this multifunctional molecule in plants under abiotic stress conditions [33,34,35]. However, as compared to other stresses, drought stress has been less studied and there is a need to have comprehensive knowledge about the exact mechanisms behind the regulation of melatonin mediated drought tolerance. Thus, the main objective of the current review is to discuss the advanced developments undertaken in the recent past which explore the melatonin-mediated drought tolerance in plants. For a better understanding of the topic, the authors started by discussing the impact of drought on plant biology, followed by an examination of the various physiological roles of melatonin. Following this, the mechanisms behind melatonin modulated physiological and molecular aspects were discussed, including photosynthetic processes, regulation of oxidative stress and other important biological mechanisms in plants under drought conditions. 

## 2. Drought Stress and its Impacts on Plant Physiology

Plants growing under water deficit conditions face many challenges at the biochemical and molecular level which ultimately causes hindrance to plant’s growth and yield [36,37]. Drought stress causes a decline in photosynthesis by disturbing the mechanism of light harvesting and its utilization, negatively impacting the metabolism of photosynthetic pigments, which declines the RuBisCo function accompanied by disruption of the photosynthetic apparatus [38,39,40]. Disruption of the chloroplast structure also negatively affects photosynthetic performance of plants growing under drought conditions [6,30]. Drought negatively influences the intercellular CO_2_ levels which favors the generation of reduced photosynthetic electron transport constituents, resulting in enhanced generation of ROS, which causes disruption of the photosynthetic apparatus [41]. Disruption of the photosynthetic apparatus due to drought leads to a reduction of the photosynthetic rate, stomatal conductance, transpiration rate, photochemical efficiency of PSII and photosynthetic electron transport rate [6,29,42]. Stomatal closure during water deficit conditions is regulated by the enhanced endogenous levels of abscisic acid (ABA), which acts as a signaling molecule to modulate a cascade of various physiological and molecular processes. This enhanced biosynthesis of ABA is due to the up regulation of the ABA biosynthetic gene *NCED3* (9-*cis*-epoxycarotenoid dioxygenase 3) [43,44]. The expression of histone H1-S is enhanced under drought stress and this protein promotes the closure of stomata [45,46]. Moreover, ABA also acts as primary messenger for cell signaling pathways which further accelerates the generation of ROS, followed by increased accumulation of cytosolic Ca^2+^ which acts as secondary messenger, stimulating other signaling cascades to regulate plant processes at the molecular level [43]. 

Drought stress decreases water potential and the relative water content of plants [47,48,49]. Declined water potential further causes a reduction in the uptake of various essential minerals, such as nitrogen, phosphorous and potassium [37,50]. Water deficit conditions have a negative impact on the nitrogen transporters and nitrogen metabolism. It is due to the down-regulation of genes, such as *AMT* (ammonium transporter), *NRT* (nitrate transporter), *NR* (nitrate reductase), *NiR* (nitrite reductase), *GS* (glutamine synthetase) and *GOGAT* (glutamate synthase), under drought stress [51]. Reduced nutrient uptake is also accompanied by reduced efficiency of their translocation to the target sites in plants growing under water deficit conditions [52]. Moreover, declined root growth in soils having low water also negatively affects the efficiency of the nutrient uptake [53]. 

**Figure 1:**
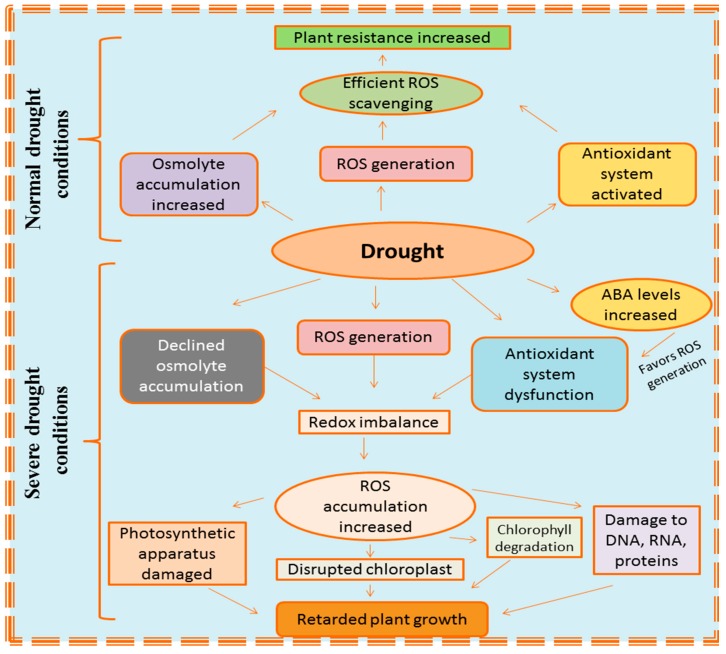
Diagrammatic sketch explaining various responses of plants under drought conditions.

Drought stress causes an imbalance between the production of ROS and their scavenging, leading to oxidative stress in plant cells [36]. This ROS scavenging failure leads to the over accumulation of ROS in plant cells, resulting in oxidation of proteins, peroxidation of lipid membranes and damage to genetic material [54,55]. Increased ROS levels also cause a reduction in fixation efficiency of CO_2_ accompanied by enhanced photorespiration [56]. To counterattack the negative effects of drought induced oxidative stress, plant’s enzymatic and non-enzymatic antioxidants work together to provide drought resistance [12,36]. However, severe drought causes an imbalance in redox homeostasis, which is mainly due to the declined efficiency of the anti-oxidative defense system. For example, activities of enzymes, such as catalase (CAT) and ascorbate peroxidase (APX), were reported to decrease under high drought conditions [57]. The production of various osmolytes, such as soluble sugars, proline and glycine-betaine, also become enhanced, resulting in more accumulation of these compounds which act as osmoprotectants under drought stress [58,59]. These osmolytes assist in maintaining the leaf turgor which results in efficient stomatal conductance followed by better CO_2_ intake by leaves and water uptake by roots [60,61]. The enhanced osmoprotectant accumulation is due to the up-regulation of genes involved in biosynthesis of osmolytes, aquaporins, LEA proteins, accompanied by regulation of various important transcription factors [45]. Figure 1 gives an overview on various responses of plants under drought stress.

## 3. Role of Melatonin in Regulation of Plant Physiology

Melatonin is a growth regulator known for its important roles in the regulation of plant growth and development [62,63]. It regulates plant’s developmental processes starting from the seed germination and has been considered to show similar effects, such as auxins, during the process of etiolation [64]. It is also believed that melatonin and auxins can have a co-regulatory impact on plant growth [65]. The concentration of melatonin also acts as a rate limiting step in the regulation of physiological processes. At lower concentrations, it promotes the growth, whereas at higher concentrations, it has inhibitory effects [66]. Melatonin also regulates the important plant processes such as morphogenesis, rhizogenesis and caulogenesis [67,68]. Melatonin positively regulates the growth of roots. This was supported by studies carried out on genetically modified rice in which gene encoding serotonin-N-acetyltransferase (SNAT) was over expressed. The over expression of *SNAT* resulted in a manifold enhancement of endogenous melatonin levels accompanied by a significant increment of rice seedling root growth [69]. In plant roots, melatonin also induces the formation of root primodia from pericycle cells [70,71,72,73]. Transcriptomic studies carried out on cucumber roots established that melatonin treatment up-regulated 121 genes, and down-regulated 196 genes. This transcriptomic regulation enhanced the plant growth by increasing the total count of lateral roots [74]. This melatonin mediated root growth in plants is supposed to be regulated in an auxin dependent manner [75,76].

Melatonin also promotes plant growth by enhancing the efficiency of carbon assimilation [77,78]. Moreover, another fact favoring the improved photosynthesis is the stimulated stomatal conductance after melatonin application [6]. Photochemical efficiency of PSII is also stimulated by melatonin, enhancing the overall photosynthesis [42,79]. Moreover, melatonin also boosts the accumulation of RuBisCO along with enhanced total nitrogen and protein content [80]. Melatonin mediated enhancement in photosynthesis is also accompanied by the reduced catabolism of chlorophyll molecules and down-regulation of genes favoring the process of senescence [81]. A delay in senescence in melatonin treated plants is favored by low H_2_O_2_ levels accompanied by high APX activity. Additionally, melatonin regulates the ascorbate-glutathione cycle, resulting in more accumulation of ascorbate and glutathione, accompanied by low levels of dehydroascorbate and oxidized glutathione [82]. Seeds treated with melatonin before sowing resulted in improved germination and vigor plants [77,83]. This melatonin seed priming has been followed by overall better vegetative and reproductive growth of plants leading to improvement in yields [84,85]. In addition to other physiological processes, melatonin also regulates fruit ripening. In tomatoes, melatonin has been observed to trigger fruit ripening by stimulating ethylene biosynthesis accompanied by the up-regulation of transcripts involved in ethylene signalling pathways [86]. Moreover, melatonin also regulates the biosynthesis of anthocyanin and proteins related to the process of fruit ripening [87].

## 4. Melatonin Mediated Regulation of Plant Biology under Drought Stress

### 4.1. Regulation of Photosynthetic Response

Melatonin protects the photosynthetic apparatus from the deleterious effects of drought, resulting in the recovery of photosynthetic efficiency of plants [6,51]. Melatonin prevents the degradation of the chlorophyll molecule during drought stress and improves the photosynthesis, transpiration and stomatal conductance [51,88]. Chlorophyll degradation is catalyzed by enzymes such as, chlorophyllase (Chlase), pheophytinase (PPH) and chlorophyll degrading peroxidase (Chl-PRX) [11,89,90,91,92,93]. The reduction in degradation of chlorophyll after melatonin treatment is due to the down-regulation of genes including *Chlase, PPH* and *Chl-PRX* [11]. Additionally, melatonin also recovers the content of photosynthetic accessory pigments, such as carotenoids under drought stress [31]. Another enzyme, pheophorbide-a-oxygenase (PAO), is involved in the chlorophyll metabolism. Melatonin down-regulates the transcript levels of *PAO*, resulting in reducing the rate of chlorophyll degradation under drought conditions [32]. 

Enhanced photosynthetic rate by melatonin is accompanied by improved photochemical efficiency (Fv/Fm) of photosystem II (PSII) along with a better photosynthetic electron transport rate (ETR) [31,70]. Non photochemical quenching is enhanced under drought stress and has a negative impact on photosynthetic efficiency. However, melatonin application to drought stressed plants helps in recovering photosynthetic performance [32]. The enlargement of the leaf area in melatonin treated plants provides another reason for the better photosynthetic efficiency under water deficit conditions [29]. 

The main reason behind the melatonin mediated improvement of photosynthesis in drought stress is that melatonin protects the chloroplast structures in leaves from oxidative damage [6,30]. During water deficit conditions, the length of chloroplast decreases gradually, which is accompanied by a disruption of the membrane, stroma lamellae, grana and thylakoids. However, melatonin treatment prevents all these ill effects of drought on the chloroplast structure [6]. The better relative water content in drought stresses leaves after melatonin treatment which further favors the protection of chloroplast structures [94]. The better water potential in melatonin treated plants under drought stress [29] can also aid in chlorophyll protection. Moreover, the length of stomata, and the recovery in the shape of palisade tissue accompanied by less damage to spongy tissue cells also contributes towards improved photosynthesis of drought stressed plants after melatonin treatment [6]. Additionally, melatonin also aids in maintaining the cell turgor which enhances the capacity of stomatal opening and conductance [6]. This increased stomatal conductance helps in better movement of water and CO_2_, and ultimately favors the photosynthesis in melatonin treated plants [30]. All these processes are further favored by melatonin regulated water balance in the mesophyll cells along with their turgor pressure regulation [30,95]. Moreover, it has also been suggested that in *Malus*, melatonin down-regulated a key gene of ABA biosynthetic pathway (*MdNCED3*) and simultaneously up-regulated the transcript levels of genes involved in degradation of ABA (*MdCYP707A1* and *MdCYP707A1*). This molecular response was accompanied by an anti-oxidative response and efficient scavenging of H_2_O_2_. Both of these mechanisms are believed to work in a synergistic manner to result in better stomatal function [96]. Recently, it has been established that melatonin regulated the carbon fixation pathway at a molecular level, which resulted in the recovery of the photosynthetic performance of plants growing under drought stress [31]. Melatonin up-regulates the transcript levels of various key enzymes of the carbon fixation pathway, such as *RUBISCO* (ribulose bisphosphate carboxylase), *PGK* (phosphoglycerate kinase), *GAPA* (glyceraldehyde-3-phosphate dehydrogenase), *FBA* (fructose-bisphosphate aldolase), *FBP* (fructose-1,6-bisphosphatase), *TIM* (triosephosphate isomerase), *SEBP* (sedoheptulose-1,7-bisphosphatase), *TKT* (transketolase), *RPI* (ribose 5-phosphate isomerase) and *RPK* (phosphoribulokinase) [31]. Table 1 summarizes the effect of melatonin on photosynthetic parameters of plants under drought stress. 

### 4.2. Regulation of Oxidative Stress and Antioxidative Defense System

#### 4.2.1. Impact on ROS Accumulation

Melatonin protects plants from the ill effects of drought induced oxidative stress by enhancing the ROS scavenging efficiency. This triggered ROS scavenging is due the melatonin stimulated anti-oxidative defense system of plants growing under drought conditions [6,29,30,32]. The drought induced generation of superoxide anions in plant cells is controlled by melatonin, either by enhancing the scavenging or by controlling the production of superoxide anions [6,98,99]. Moreover, scavenging efficiency of H_2_O_2_ is also enhanced by melatonin in plants growing under drought stress [6,70,96]. This is followed by enhanced detoxification of harmful hydroxyl radicals and other aldehydes involved in the induction of oxidative stress [27,70]. Melatonin also regulates the ascorbate-glutathione cycle and triggers the direct scavenging of ROS, such as H_2_O_2_ [32]. Melatonin mediated efficient ROS scavenging in the plants under drought stress leads to protection of plant cell walls. This fact is supported by the reduced levels of MDA content and decline in electrolyte leakage in melatonin treated plants under water deficit conditions [11,27,31]. 

Due to water scarcity in plant cells, biosynthesis of ABA is enhanced, resulting in accumulation of more ABA than the normal conditions. These enhanced ABA levels favor the generation of ROS, causing oxidative stress in terms of lipid peroxidation, electrolyte leakage and cause breakdown of chlorophyll molecules [100]. However, molecular studies on melatonin treated plants under drought stress revealed that a reduction in ROS levels was also accompanied by declined ABA accumulation. This declined ABA concentration was due to the melatonin mediated down-regulation of genes responsible for ABA biosynthesis and simultaneously up-regulation of genes involved in ABA catabolism [96]. Moreover, it is also believed that melatonin regulates the scavenging/generation of ROS via CK-signaling and both of melatonin and CK work synergistically to regulate drought induced oxidative stress in plant cells [11]. All of these above mentioned facts were further supported by studies in which the overexpression of *TaCOMT* (gene involved in melatonin biosynthesis) in *Arabidopsis* were subjected to water deficit conditions. In comparison to non-transgenic plants, overexpressing this gene resulted in enhanced endogenous levels of melatonin accompanied by a reduction in lipid peroxidation under drought stress [101]. Similarly, overexpression of another melatonin biosynthetic gene *MzASMT* (cloned from *Malus zumi*) in *Arabidopsis* plants grown under water deficit conditions, resulted in enhanced scavenging and better drought tolerance [102]. The impact of melatonin on various oxidative stress markers has been summarized in Table 2.

#### 4.2.2. Impact on Enzymatic and Non-Enzymatic Anti-oxidative Defense System

Due to drought stress, generation of ROS takes place in plant cells. To regulate the level of ROS, plant’s internal defense system (enzymatic and non-enzymatic) gets stimulated. Furthermore, melatonin also triggers this defense system and enhances the scavenging harmful ROS, leading to a reduction in drought induced oxidative stress [27,31]. Melatonin is considered as a multifunctional antioxidant and is a receptor-less scavenger of harmful free radicals [62]. Moreover, melatonin also acts as a stimulator of the enzymatic anti-oxidative defense system, resulting in protection of plants against oxidative damages [42,104].

In drought stressed plants, melatonin promotes activities of ABA degrading enzymes along with H_2_O_2_ scavenging enzymes like CAT, POD and APX [96]. This enhanced activity of above mentioned enzymes results in the decline of H_2_O_2_ in guard cells, indicating a direct involvement of melatonin in scavenging of H_2_O_2_ [96,105,106]. In drought stressed plants, melatonin also enhances the activities of other enzymatic anti-oxidative enzymes, such as SOD, GPX, GR, DHAR and MDHAR [27,29,32]. 

**Figure 2:**
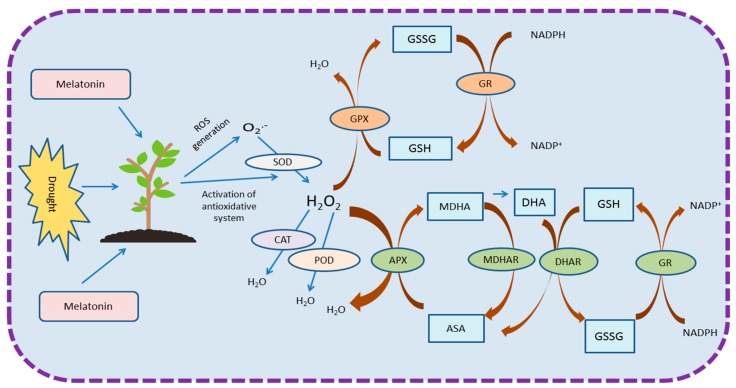
Diagrammatic explanation of the melatonin regulated anti-oxidative system and scavenging of reactive oxygen species. This diagram is a conclusion of various studies mentioned in Table 3. Abbreviations – ASA, ascorbate; APX, ascorbate peroxidase; CAT, catalase; DHA, dehydroascorbate; DHAR, dehydroascorbate reductase; GPX, glutathione peroxidase; GSH, glutathione; GSSG, oxidative glutathione; GR, glutathione reductase; H_2_O_2_, hydrogen peroxide; MDHA, monodehydroascorbate; MDHAR, monodehydroascorbate reductase; NADPH, reduced nicotinamide adenine dinucleotide phosphate; NADP, nicotinamide adenine dinucleotide phosphate; O_2_^.-^, superoxide anion; POD, peroxidase; SOD, superoxide dismutase.

Melatonin mediated ROS scavenging is controlled in drought stressed plants via the regulation of the key enzymatic cycle known as the Asada-Halliwell pathway [98]. Additionally, melatonin also regulates the AsA-GSH cycle which plays an important role in ROS detoxification. This cycle is regulated by enzymes, such as APX, MDHAR, DHAR and GR [30,32]. In chloroplast, GR is responsible for AsA homeostasis [107], and in drought stressed plants, melatonin up-regulates the GR activity [30]. Another enzymatic antioxidant, GPX, has capability to scavenge hydroperoxides, H_2_O_2_ and lipid peroxides, and under drought conditions, melatonin up-regulates the activity of GPX, resulting in efficient ROS scavenging [30,108]. Figure 2 explains the melatonin regulated enzymatic anti-oxidative defense system in plants growing under drought stress.

Table 3 summarizes the effects of exogenous applied melatonin on the enzymatic antioxidants in plants growing under water deficit conditions.

The regulation of AsA-GSH cycle by melatonin under drought stress results in the enhancement of AsA/DHA and GSH/GSSG ratios [32,98]. The enhanced activity of GR is responsible for the increased ratio of NADP^+^/NADPH followed by better performance of photosynthetic electron transport (PET). This increased PET also inhibits the generation of superoxide anions [98]. Moreover, melatonin mediated control of superoxide anion production under drought stress is also due to the fact that melatonin reduces the consumption of O_2_ flux in those conditions when ADP levels are higher [98]. Moreover, melatonin also enhances the DPPH-radical scavenging efficiency of plants growing under water deficit conditions [42]. After melatonin treatment, an increase in AsA and GSH contents in drought stressed plants is also accompanied by a reduced H_2_O_2_ content [98]. This stimulated biosynthesis of AsA and GSH is considered to be necessary for the ROS balance in plants under low water conditions [30]. It is also suggested that glutamylcysteine synthase, which is a key enzyme of GSH biosynthetic pathway [109], might have up-regulated by melatonin, but further research is required to study the exact mechanism. Additionally, AsA and GSH are also involved in the scavenging of superoxide anions, and the process is further triggered by melatonin under drought stress [6]. Proline is a non-enzymatic antioxidant which is also involved in providing resistance to plants under water deficit conditions and melatonin treatment enhances its biosynthesis, resulting in the reduction of drought induced oxidative stress [6]. Moreover, it is also suggested that proline aids in maintaining cell function by reducing the levels of ROS and stabilizing cell membranes [6]. Phenolic compounds are also a potential antioxidant and their accumulation is boosted by exogenous applied melatonin, which can be beneficial for plants growing under drought stress [89]. Melatonin also stimulates the biosynthesis of compatible solutes, such as soluble sugars, which are responsible for maintaining the turgor and osmotic pressure of plant cells growing in water deficit conditions [29]. This stimulated biosynthesis of osmolytes is a part of a mechanism for maintaining the osmotic balance of plants under drought stress [41,110]. Additionally, they also play a role in enhancing the ROS scavenging efficiency and cell wall protection against abiotic stress conditions [111,112]. The various effects of melatonin on non-enzymatic antioxidants under drought stress have been summarized in Table 4.

### 4.3. Regulation of Other Biological Processes Related to Drought Tolerance

Mitogen-activated protein kinase (MAPK) cascade pathways play a crucial role in the regulation of the plant’s biological processes under abiotic stresses, including drought [113]. Transcription factors (TFs), such as NAC, WRKY, MYB and DREB are the main components of MAPK signaling pathway in plants under stress conditions [114]. These TFs are involved in the regulation of various stress responsive genes responsible for abiotic stress tolerance [115]. Melatonin under drought stress regulates the MAPK pathway by up-regulating the expression pattern of MAPKs, such as *Asmap1* and *Aspk11*. It is accompanied by the up-regulation of key TFs, including *WRKY1, DREB2* and *MYB* [99]. This melatonin-mediated regulation of MAPK cascade is believed to be regulated via H_2_O_2_ signaling, resulting in the enhanced plant’s resistance against drought stress [99]. Drought stress in plants causes negative impacts upon the nitrogen metabolism [116,117]. Melatonin regulates nitrogen metabolism under drought stress by modulating the physiological and molecular aspects of plant biology [51]. The activities of nitrogen metabolic enzymes, such as NR, NiR, GS and GOGAT are enhanced by melatonin [51]. Furthermore, the reason behind melatonin induced activities of nitrogen metabolic enzymes is explained by the fact that melatonin also up-regulates the expression pattern of genes, including *NR, NiR, GS* and *GOGAT* in plants under water deficit conditions [51]. Additionally, transcript levels of genes involved in nitrogen uptake, *AMT* (ammonium transporter) and *NRT* (nitrate transporter) are also enhanced by melatonin in plants growing under drought stress [51]. Drought induced senescence is delayed after melatonin application, which is due to the down-regulation of gene *SAG12* (senescence associated gene 12) [32]. Furthermore, this delaying of senescence is favored by melatonin mediated overexpression of genes, such as *JUB1* and *DREB2A* under drought conditions [11]. 

Transgenic studies involving overexpression of *TaCOMT* in *Arabidopsis* revealed that melatonin up-regulated various drought responsive genes, such as *RAB18, RD29A, KIN1* and *DREB2A* [101]. Additionally, it is also suggested that melatonin provides drought tolerance by regulating GA and IAA biosynthetic pathways. Melatonin is believed to suppress IAA biosynthesis via GA-signaling accompanied by better drought resistance in plants [101]. Cytokinin (CK) biosynthesis is stimulated by melatonin by up-regulating the transcript levels of key genes involved in CK-signaling, including Type-A *RRs*, Type-B *RRs* (response regulators), *HKs* (histidine kinases) and *HPs* (histidine phosphotransferases) [11]. This melatonin mediated CK-signaling has been associated with the induction of drought resistance in plants [11].

Cuticle waxes are important for plants growing under low water conditions, as these compounds assist in controlling the water loss through the leaf’s surface [97]. Melatonin stimulates the biosynthesis of cuticular waxes and increases their deposition on the leaf’s surface, resulting in minimum water loss. This enhanced biosynthesis is due to the up-regulation of the transcript levels of genes, such as *KCS1* (ketoacyl-CoA synthase 1), *CER3* (*ECERIFERUM3),* TTS1 (triterpenoid synthase 1) and LTP1 (lipid transfer protein 1), which encodes enzymes involved in wax biosynthetic pathways [97].

## 5. Conclusions

**Figure 3:**
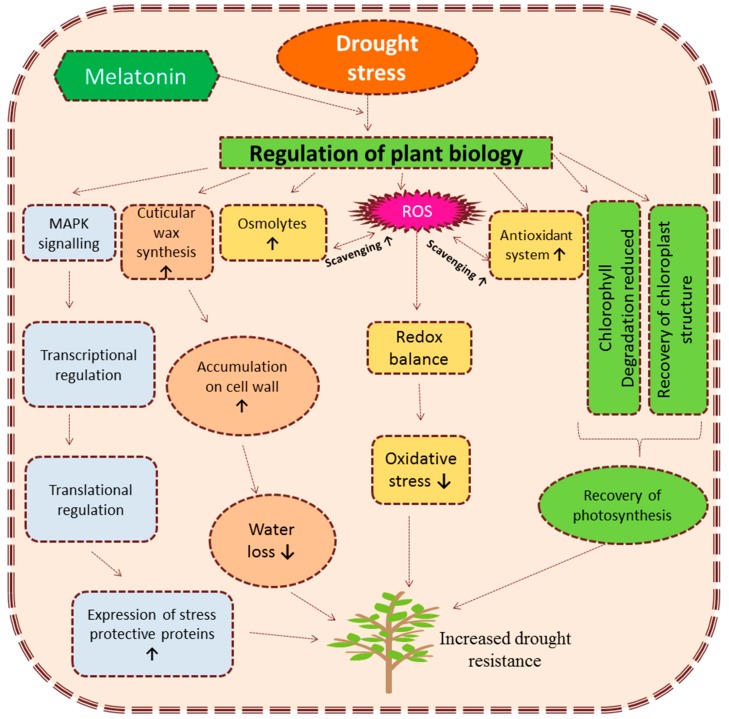
An overview of melatonin mediated regulation of drought stress in plants. ROS (reactive oxygen species), MAPK (Mitogen-activated protein kinase) ↑ = increase, ↓ decrease.

Melatonin provides resistance to plants growing under drought conditions by enhancing the scavenging of ROS. This prevents cells from oxidative damage and assists in the recovery of chloroplast structures resulting in the improvement of photosynthetic efficiency of plants. Melatonin mediated protection of drought stressed cells is regulated via stimulated cell signaling which ultimately controls various physiological aspects at a molecular level. Figure 3 provides a detailed overview of melatonin mediated regulation of plant biology under drought stress. As drought stress directly reduces the yield and quality of crops, the implication of melatonin at a field level can be helpful from an agronomic point of view. Some recent studies have also reported the enhanced drought tolerance after gene manipulation (GM) and developing genetic modified plants with better melatonin biosynthesis. This GM technology can be beneficial in developing better drought resistant varieties. Moreover, the identification of other key genes involved in providing drought resistance and studying their behavior under melatonin treatment can open new possibilities to develop drought tolerant crops. 

## Figures and Tables

**Table 1:** Summary table explaining the effect of exogenous applied melatonin on various photosynthetic parameters under drought stress.

Plant Name	Conc.	Impact on Photosynthetic Parameters under Drought Stress	Reference
*Actinidia chinesis*	100 µM	Recovery of leaf area, chlorophyll and carotenoid contents, photochemical efficiency of PSII along with photosynthetic electron transport rate. Better photosynthetic rate, stomatal conductance and transpiration rate.	[31]
*Agrostis stolonifera*	20 µM	Recovery in relative water content, chlorophyll content and photochemical efficiency. Down-regulation of genes involved in chlorophyll degradation like *CHLASE, PPH* and *CHL-PRX.*	[11]
*Coffea arabica*	300 µM	Better leaf water potential. Increased chlorophyll content, photosynthetic rate, stomatal conductance and transpiration rate.	[29]
*Cucumis sativus*	100 µM	Improved photosynthetic rate, stomatal conductance, chlorophyll content and photochemical efficiency of PSII.	[70]
*Dracocephalum moldavica*	100 µM	Recovery of chlorophyll content accompanied by increased leaf length and leaf area.	[27]
*Malus domestica*	100 µM	Recovery of chlorophyll content accompanied by down-regulation of transcript levels of chlorophyll degrading enzyme *PAO*. Improved photosynthetic rate, stomatal conductance, and photochemical efficiency of PSII along with photosynthetic electron transport rate.	[32]
		Increased chlorophyll content, photosynthetic rate, stomatal conductance and transpiration rate. Increased length, width and aperture of stomata.	[51]
*Malus prunifolia* *and* *M. hupehensis*	100 µM	Better relative water content. Improved photosynthetic rate, stomatal conductance and chlorophyll content. Recovery of stomatal opening along with improved stomatal length, width and aperture.	[96]
*Solanum lycopersicum*	200 µM	Recovery of chlorophyll content.	[88]
	100 µM	Better cell wall stability accompanied by less leaching of chlorophyll molecules.	[97]
	0.1 mM	Improved photosynthetic rate, stomatal conductance, chlorophyll content and photochemical efficiency of PSII.	[98]
*Triticum aestivum*	100 µM	Recovery of chloroplast apparatus, photosynthetic rate, stomatal conductance, transpiration rate and photochemical efficiency of PSII.	[30]
*Vitis vinifera*	100 nM	Increased chlorophyll content and photochemical efficiency. Recovery of damaged chloroplast ultrastructure and stomata.	[6]
*Zea mays*	1 mM	Recovery of photochemical efficiency of PSII.	[94]
	100 µM	Better leaf area accompanied by recovery in chlorophyll content, photosynthetic rate, stomatal conductance and transpiration rate. Improved water potential, photochemical efficiency of PSII along with photosynthetic electron transport rate.	[42]

Chlase, chlorophyllase; PPH, pheophytinase; Chl-PRX, chlorophyll degrading peroxidase, PAO, pheophorbide-a-oxygenase; PSII, photosystem II.

**Table 2:** Summary table explaining the effect of exogenous applied melatonin on various oxidative stress markers under drought stress.

Plant Name	Conc.	Impact on Oxidative Stress Markers under Drought Stress	Reference
*Actinidia chinesis*	100 µM	Reduction in MDA content and membrane injury index.	[31]
*Agrostis stolonifera*	20 µM	Reduction in contents of H_2_O_2_ and MDA accompanied by declined electrolyte leakage.	[11]
*Avena nuda*	100 µM	Reduction in contents of superoxide anion and H_2_O_2_.	[99]
*Brassica napus*	50 µM	Reduction in H_2_O_2_ content.	[103]
*Coffea arabica*	300 µM	Reduction in lipid peroxidation.	[29]
*Cucumis sativus*	100 µM	Reduction in contents of H_2_O_2_, hydroxyl radical and MDA accompanied by declined electrolyte leakage.	[70]
*Dracocephalum moldavica*	100 µM	Reduction in contents of H_2_O_2_, MDA and other aldehydes which cause oxidative stress accompanied by declined electrolyte leakage.	[2]
*Malus domestica*	100 µM	Reduction in electrolyte leakage accompanied by declined H_2_O_2_ content.	[51]
		Reduction in H_2_O_2_ content.	[32]
*Malus prunifolia* *and* *M. hupehensis*	100 µM	Reduction in H_2_O_2_ content. Accumulation of ABA is reduced.	[96]
*Solanum lycopersicum*	200 µM	Reduction in lipid peroxidation.	[88]
	0.1 mM	Reduction in contents of superoxide anion and MDA.	[98]
*Triticum aestivum*	100 µM	Reduction in contents of superoxide anion, H_2_O_2_ and MDA accompanied by declined electrolyte leakage.	[30]
*Vitis vinifera*	100 nM	Reduction in contents of superoxide anion and H_2_O_2_.	[6]
*Zea mays*	100 µM	Reduction in contents of H_2_O_2_ and MDA. Better DPPH scavenging activity.	[42]

ABA, abscisis acid; DPPH, 2,2-diphenyl-1-picryl-hydrazyl-hydrate; H_2_O_2_, hydrogen peroxide; MDA, malondialdehyde.

**Table 3:** Summary table explaining the effect of exogenous applied melatonin on various anti-oxidative enzymes under drought stress.

Plant Name	Conc.	Impact on Antioxidative Enzymes under Drought Stress	Reference
*Avena nuda*	100 µM	Enhanced activities of APX, CAT, POD and SOD.	[99]
*Brassica napus*	50 µM	Enhanced activities of APX, CAT and POD.	[103]
*Coffea arabica*	300 µM	Enhanced activities of APX and CAT, but no significant difference in SOD activity.	[29]
*Cucumis sativus*	100 µM	Enhanced activities of CAT, POD and SOD.	[70]
*Dracocephalum moldavica*	100 µM	Enhanced activities of APX, CAT, GPX and SOD.	[27]
*Malus domestica*	100 µM	Enhanced activities of APX, CAT, POD, DHAR, MDHAR and GR.	[32]
*Malusprunifolia* and *M. hupehensis*	100 µM	Enhanced activities of APX, CAT and POD.	[96]
*Solanum lycopersicum*	200 µM	Enhanced GR activity.	[88]
	0.1 mM	Enhanced activities of APX, CAT, GR, POD and SOD.	[98]
*Triticum aestivum*	100 µM	Enhanced activities of APX, GPX, DHAR, MDHAR, GST and GR. Up-regulation in the transcript levels of *APX, DHAR, MDHAR4, GPX, GPX1, GR* and *GST2.*	[30]
*Vitis vinifera*	100 nM	Enhanced activities of CAT, POD and SOD.	[6]
*Zea mays*	100 µM	Enhanced activities of APX, CAT, POD and SOD.	[42]

APX, ascorbate peroxidase; CAT, catalase; DHAR, dehydroascorbate reductase; GPX, glutathione peroxidase; GR, glutathione reductase; GST, glutathione-S-transferase; MDHAR, monodehydroascorbate reductase; POD, peroxidase; SOD, superoxide dismutase.

**Table 4:** Summary table explaining the effect of exogenous applied melatonin on various non-enzymatic antioxidants and osmotic adjustments under drought stress.

Plant Name	Conc.	Impact on Non-Enzymatic Antioxidants under Drought Stress	Reference
*Actinidia chinesis*	100 µM	Accumulation of soluble sugars and proline is increased. This is accompanied by better cellular osmotic adjustments, resulting in reduction of cell injury.	[31]
*Brassica napus*	50 µM	Increased accumulation of total soluble sugars and proline, accompanied by better osmotic regulation capacity.	[103]
*Coffea arabica*	300 µM	Increased accumulation of sucrose, total soluble sugars, ascorbate and proline, accompanied by improvement in leaf water potential.	[29]
*Dracocephalum moldavica*	100 µM	Increased accumulation of proline accompanied by better relative water content.	[27]
*Malus domestica*	100 µM	Increased accumulation of GSH, total GSH, AsA and total AsA. Reduction in DHA and GSSG accumulation, accompanied by higher ratios of GSH/GSSG and AsA/DHA.	[32]
*Solanum lycopersicum*	200 µM	Accumulation of p-coumaric acid (a phenolic compound) is increased under only melatonin treatment.	[88]
	0.1 mM	Increased accumulation total AsA.	[98]
*Triticum aestivum*	100 µM	Increased accumulation of GSH, total GSH, AsA and total AsA. Reduction in DHA accumulation.Higher ratios of GSH/GSSG and AsA/DHA.Better cell turgor accompanied by improved water holding capacity leads to osmotic adjustments in drought stressed cells.	[30]
*Vitis vinifera*	100 nM	Accumulation of ascorbate, glutathione and proline is enhanced. Proline is suggested to be involved in regulation of osmotic potential of drought stressed cells.	[6]

AsA, ascorbate; DHA, dehydroascorbate; GSH, glutathione; GSSG, oxidative glutathione.
